# First-in-human cases and preclinical experience of a novel ICE catheter

**DOI:** 10.3389/fcvm.2024.1406470

**Published:** 2024-09-10

**Authors:** Guangan Liu, Jingjing Wu, Fang Fang, Weipeng Zhao, Minmin Sun, Jihong Zhang, Simeng Liu, Mimi Wang, Feng Liu

**Affiliations:** ^1^ Cardiology Department, Suzhou Kowloon Hospital, Shanghai Jiao Tong University School of Medicine, Suzhou, China; ^2^ Department of Echocardiography, Fuwai Hospital, CAMS and PUMC, Beijing, China; ^3^ Department of Echocardiography, Zhongshan Hospital of Fudan University, Shanghai, China; ^4^ Department of Medical, ICE Intelligent Healthcare Co., Ltd, Suzhou, China

**Keywords:** intracardiac echocardiography (ICE), paroxysmal supraventricular tachycardia (PSVT), atrial fibrillation (AF), image quality, endocardial damage

## Abstract

**Introduction:**

The primary objective of our study was to evaluate the first use of a novel intracardiac echocardiography (ICE) catheter in human subjects. This study aimed to assess its practicality, image clarity, and guidance role during electrophysiology procedures.

**Methods:**

Two patients underwent procedures using the novel ICE catheter. Post-procedure evaluations were conducted by four operators, who assessed the imaging quality and overall performance of the catheter. Anatomical and blood test results were also analyzed to determine the safety and impact on internal cardiac structures.

**Results:**

Both patients were discharged one day after the procedure without any complications. The novel ICE catheter provided comparable imaging quality to existing commercial catheters. The catheter's advanced design allowed for detailed imaging at short distances, essential for accurate diagnosis and treatment planning. Moreover, it successfully navigated complex anatomical structures like the atrial septum and left atrial appendage.

**Discussion:**

These preliminary studies indicate that the novel ICE catheter achieves a level of safety and effectiveness comparable to previously available commercial catheters. The findings highlight its potential to meet current clinical needs, particularly in sophisticated anatomic interventions. Despite the prolonged thrombin time after anticoagulant administration, both types of ICE catheters were non-damaging to cardiac structures during routine operations. The study underscores the importance of using trans-septal large inner diameter sheaths to minimize complications when advancing the catheter into the left atrium.

## Background

Since the 1960s, the use of intracardiac echocardiography (ICE) catheters has been showcased globally. Notably, in 1981, Glassman and Kronzon made a significant contribution by embedding a 7.5 MHz ultrasonic into a flexible coaxial cable, which was then utilized in 20 patients (1). Although the prototype emerged early, technological advances in ICE have not kept pace with those of its peers. Furthermore, it's noteworthy that ultrasound imaging is the only major medical imaging modality for which no one has been awarded a Nobel Prize, since the development depending on many prior insights from physics (2). In 2000, the first phased-array AcuNAV 10 French (10F) catheter (Johnson and Johnson MedTech) was introduced to global market, and since then, it has maintained a dominant position for more than 20 years.

With the onset of the new century, rapid developments in fields such as structural heart and electrophysiology diseases have propelled ICE into new applications (3–6). The demand of visualizing small cardiac structures, including valves, left atrial appendage and left atrial wall, has driven the adoption of 3D and 4D ICE (6–9). In comparison to the well-stablished transesophageal echocardiography (TEE), ICE presents unique advantages, offering high spatial resolution for imaging at short distances and eliminating the need for general anesthesia (8, 10, 11). Despite its cost disadvantage, ICE has demonstrated its value in various intervention procedures.

Meanwhile, besides the AcuNAV and ViewFlex ICE (Abbott Laboratories) catheters, only one domestic product received approval in China by the end of 2023. However, the domestic device currently lacks substantial supporting evidence. On the other hand, regulations governing medical insurance costs and the lack of 10 F and larger long sheaths in the local market have led interventionists to position ICE catheters mostly in the right atrium. In exceptional cases, they may choose an off-label approach, delivering the probe directly to the left atrium through the small hole formed by 8 F puncture systems. Such off-label usage entails additional risks.

**Figure 1:**
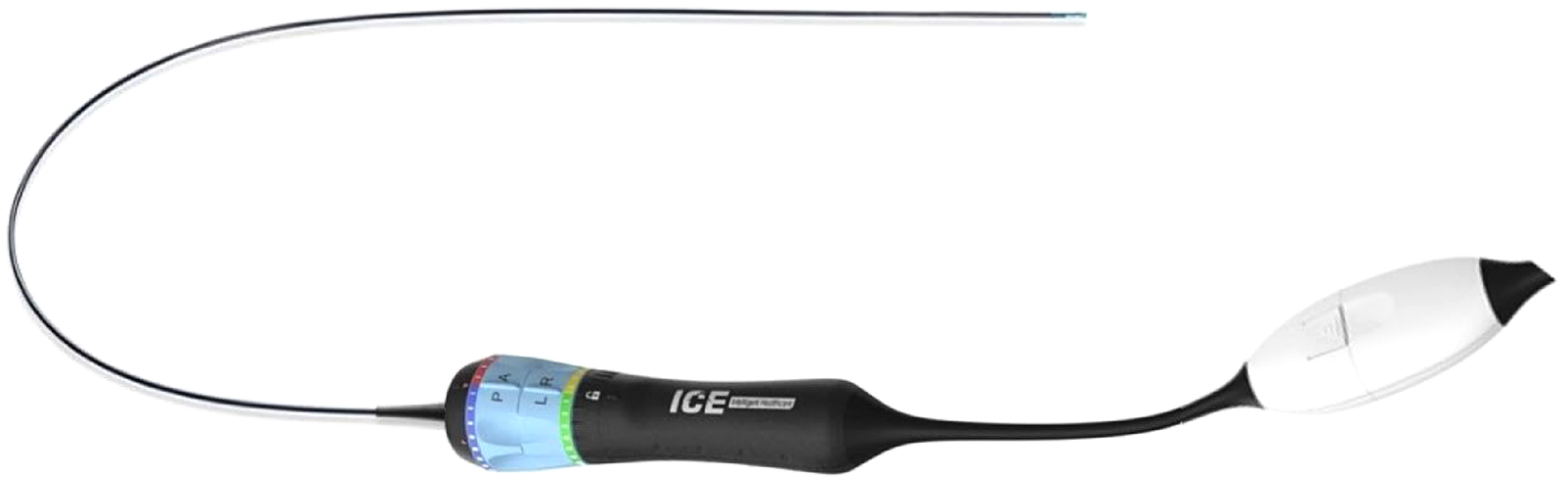
Arch echo™ ICE probe. The novel ICE catheter operates at a frequency range of 6.5 ± 0.5 MHz, is designed for ease of use with standard electrophysiology lab equipment, and features a streamlined, smooth surface to minimize tissue trauma.

We have developed a novel 10 F, 4-directional steerable ICE probe (Arch Echo™, ICE Intelligent Healthcare Co., Ltd) equipped with a 64-element imaging matrix, enabling real-time imaging during various procedures (Figure 1). To verify its safety and effectiveness, we conducted the First-in-Human (FIH) and preclinical *in vitro* tests. The FIH study and animal tests have been approved by the corresponding institutional review board.

### Preclinical vitro tests protocol

In the acute *in vitro* testing, the experimental porcine models were divided into a test group (*n* = 4, Arch Echo™) and a control group (*n* = 2, AcuNAV 10F). Additionally, one subject was used to assess the AcuNAV 10F catheter's ability to cross the septum without a sheath. The main purpose was to simultaneously verify the effectiveness and safety of the large-bore ICE catheter for off-label operations in clinical practice. The data was collected for further development.

After an overnight fast, all porcine subjects were induced and subsequently mechanically ventilated with isoflurane. Neuromuscular paralytics were not administered. Bilateral venous access was achieved percutaneous and/or surgical cutdown. Two 10 F short sheaths, either Cordis or Terumo, were placed. If a trans-septal puncture was planned, the systemic heparinization was administered. Cardiac structure images were successfully collected in all animals. Subsequently, all six pigs underwent euthanasia for heart anatomy examination, and blood tests were conducted on last animal from each group (Table 1).

**Table 1:** Animal cardiac structures and ultrasound modes.

Cardiac Structures	Ultrasound modes^a^
Right atrium	B, Color, PW, CW, M
Tricuspid valve	
Right ventricular	
Right ventricular outflow tract	
Atrial septum	
Left atrium	
Mitral valve	
Left atrial appendage	
Pulmonary vein	
Left ventricular	
Aortic valve	
Others	

^a^ B mode, brightness mode, a two-dimensional ultrasound image; PW, pulsed-wave Doppler; CW, continuous-wave Doppler; M mode, motion mode, an image displayed with the abscissa representing time, and the ordinate showing distance from the transducer.

### Image quality score principles

The image quality scores were independently recorded by two cardiac sonographers (Z. WP and S. MM). In instances of discrepancies, a third sonographer (F. F) would arbitrate and make the final decision. Results meeting clinical diagnostic requirements should have an average score per site not less than 3.

The sonographers would grade ICE images according to the scoring criteria described in Table 2.

**Table 2:** ICE image scoring criteria.

Point	Reference
5	Excellent image quality that fulfills clinical diagnostic and treatment needs, very satisfactory
4	Good image quality that meets clinical diagnostic and treatment needs, satisfactory
3	The image has flaws but meets clinical needs, generally satisfactory
2	Poor image quality interferes with clinical diagnosis and treatment, semi-satisfactory
1	Poor image quality prevents completion of clinical diagnosis and treatment, unsatisfactory
Mode	Reference
B	Penetration, contrast resolution, spatial resolution, temporal resolution, field uniformity, noise suppression
Color	Penetration, sensitivity, spatial resolution, temporal resolution, hemodynamics, noise suppression
PW	Penetration, sensitivity, spectral dynamic range or level, sound
CW	Sensitivity, spectral dynamic range or level, sound
M	Penetration, temporal resolution

### Preclinical tests results

**Figure 2:**
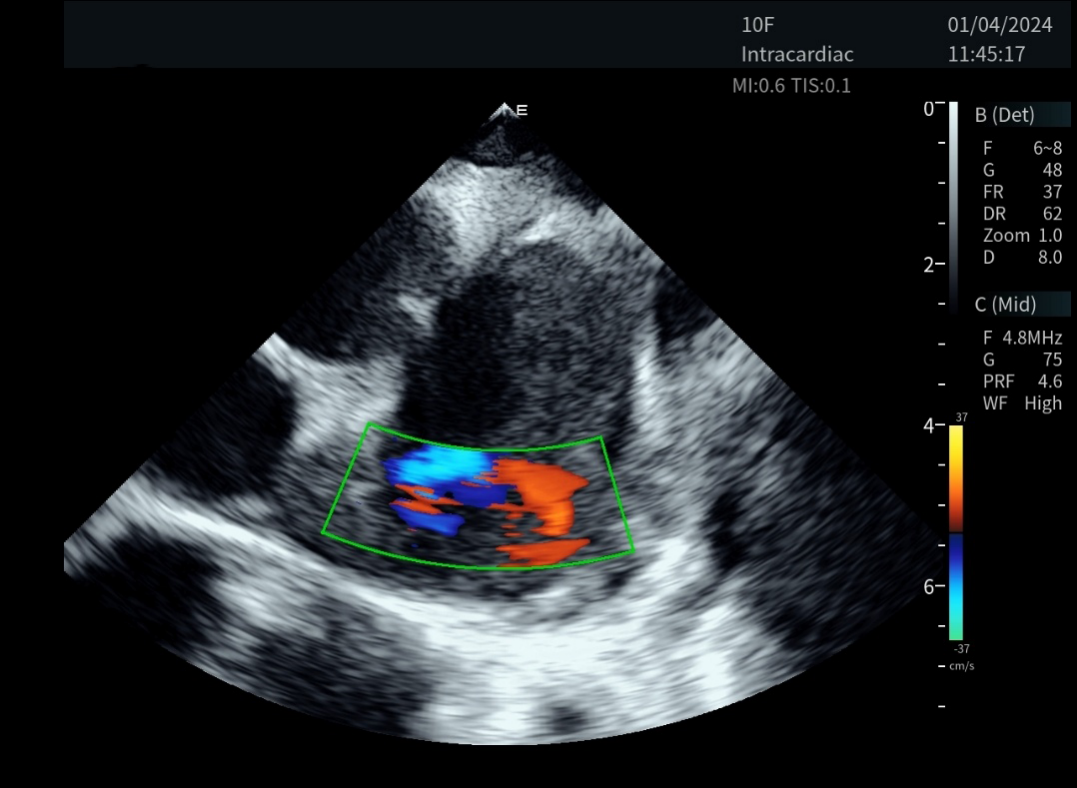
Collection of preclinical assessment data.

We conducted comprehensive preclinical assessments using porcine models. The data collection included detailed imaging of cardiac structures, evaluation of endocardial damage, and scoring by experienced intervention cardiologists (Figure 2). The preclinical experiment was conducted by a team of two intervention cardiologists, each with specific roles: the primary operator, who conducted the catheter placement and electrophysiology procedures; and the secondary operator, assisted with equipment handling and monitoring. The porcine models included five boars and just one sow in the experimental group. The average weight was 51.37 Kg. All animals completed the procedures, and no complication occurred during or after the operations. All images of various cardiac structures using the ICE catheter in different modes were collected for later scoring.

The novel ICE catheter recorded color doppler ultrasound images of the left atrial appendage.

**Figure 3:**
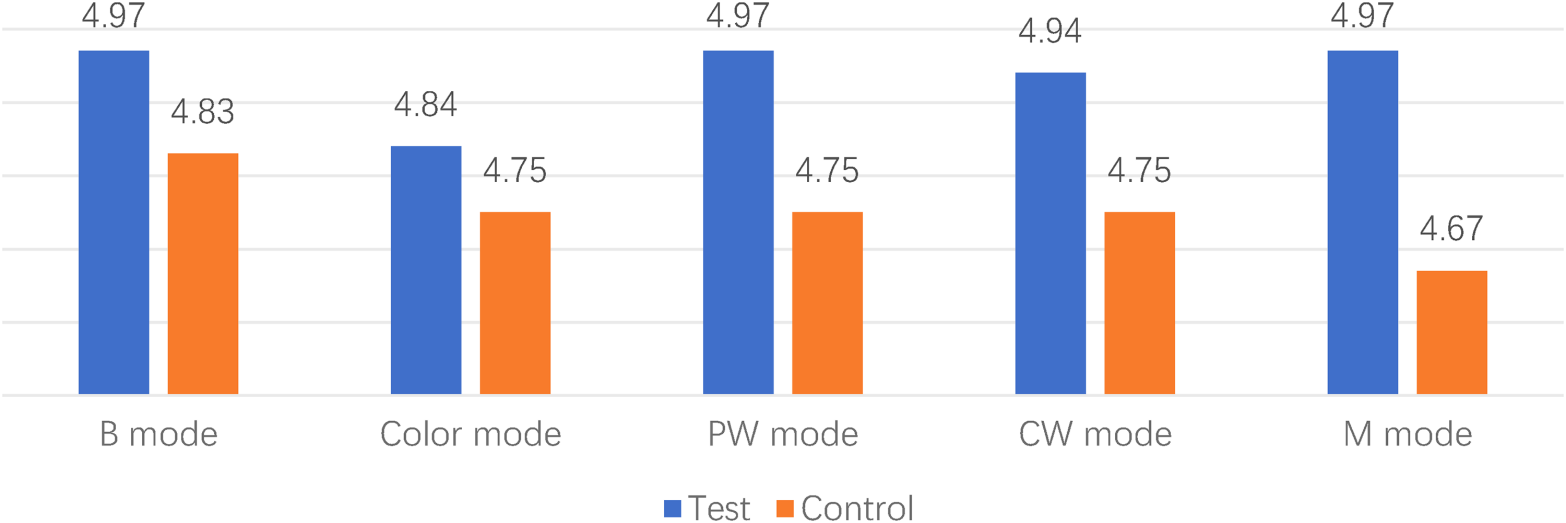
Comparison between different modes.

**Figure 4:**
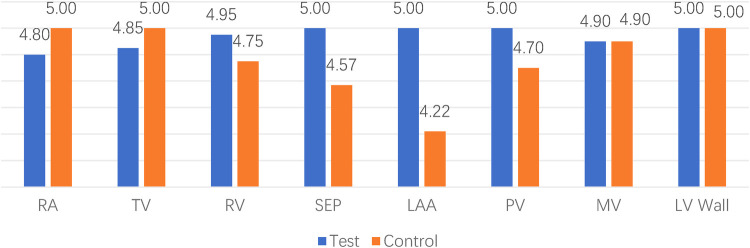
Comparison between different locations. RA indicates right atrium; TV, tricuspid valve; RV, right ventricular; SEP, atrial septum; LAA, left atrial appendage; PV, pulmonary vein; MV, mitral valve; LV wall, left ventricular wall.

The majority of videos scored 3 points or higher across various ultrasound modes and structures. However, in the case of the 1st animal using the control device, a sonographer (S. MM) scored 2 points for the color mode of the right atrium and pulmonary veins, indicating that the image quality may not meet clinical requirements. Additionally, all scores showed similarity, ranging from 3 to 5, suggesting that the two set of images were at the similar levels (Figures 3, 4).

The last animal from each group underwent pre- and post-procedure blood tests, including blood routine, biochemistry, and coagulation function assessments. This was a safety verification indicator used to evaluate whether the blood parameters of the ICE catheter have changed before and after it enters the animal's body. These last two animals from each group were selected to mitigate the learning curve. In Table 3, the findings indicated that both the pre- and post-procedure examinations for the final animal in each group fell within the normal range, except for the thrombin time (TT), which unavoidably prolonged following the administration of anticoagulant medications.

**Table 3:** Blood tests before and after the procedures in the test and control group.

Parameters		Test group		Control group	
Name	Biological ref range^a^	Preprocedure	Postprocedure	Preprocedure	Postprocedure
RBC ^a^10^12/L	5.50–9.00	6.96	7.02	4.41	4.66
Haemoglobin g/L	100–160	99	101	87	95
WBC ^a^10^9/L	11–22	13.52	18.84	15.66	15.1
Neutrophils ^a^10^9/L	2.80–16.10	3.79	9.36	4.37	4.18
Lymphocytes ^a^10^9/L	4.80–16.20	8.25	8	6.87	6.33
Monocytes ^a^10^9/L	0.20–2.25	0.94	1.35	3.87	4.26
Platelets ^a^10^9/L	200–500	388	243	384	317
Blood biochemistry					
ALT U/L	22–47	33.9	31	21.2	19.6
AST U/L	15–55	16.7	19.1	50.2	55.9
ALP U/L	41–176	81.4	78.1	30.2	28.9
GGT U/L	31–52	39.3	32.8	24.8	33.3
Creatinine umol/L	70.72–203.32	91.8	93.4	100.3	89.6
UREA mmol/L	2.9–8.8	2.66	3.12	3.16	3.72
Coagulation test					
INR		1.01	1.16	1.14	1.23
Prothrombin times	9.20–15.00	12.07	13.99	13.75	14.94
Thrombin times	10.00–150.00	25.39	>90	28.9	>90
APTTs	21.00–200.00	14.0	83.47	20.54	38

RBC indicates red blood cell; WBC, white blood cell; ALT, alanine aminotransferase; AST, aspartate aminotransferase; ALP, Alkaline phosphatase; GGT, gamma-glutamyl transferase; UREA, blood urea nitrogen; and INR, international normalized ratio.

^a^ The reference range is based on the porcine model.

**Table 4:** Inclusion and exclusion criteria.

Inclusion criteria
1. Patients undergoing cardiac interventional procedures for arrhythmia radiofrequency ablation and/or left atrial appendage closure.
2. Age ≥** **18 years,** **≤** **80 years, of any gender.
3. Subjects themselves or their guardians must possess the ability to comprehend the purpose of this study, demonstrate adequate compliance with the protocol, and sign the informed consent form.
Exclusion criteria
1. Thrombus in the cardiac chamber.
2. History of myocardial infarction or presence of severe coronary artery lesions requiring intervention confirmed by coronary imaging within 3 months.
3. History of cardiac surgery such as artificial valve replacement, atrial septal defect closure, and heart transplantation, which makes the patient unsuitable for further cardiac interventional procedures.
4. Active infection, including but not limited to fever, cough, sputum, chest tightness and abdominal pain.
5. Other combined serious diseases, including but not limited to severe hypotension, shock, pulmonary insufficiency, and malignant tumor.
6. Left ventricular ejection fraction (LVEF)** **<** **35%.
7. New York Heart Association (NYHA) class III or IV, and not yet corrected.
8. Participation in other clinical trials and not yet completed.
9. Other specific circumstances identified by the investigators where the subject is deemed unsuitable for enrollment.

### Endocardial damage caused by ICE catheter

**Figure 5:**
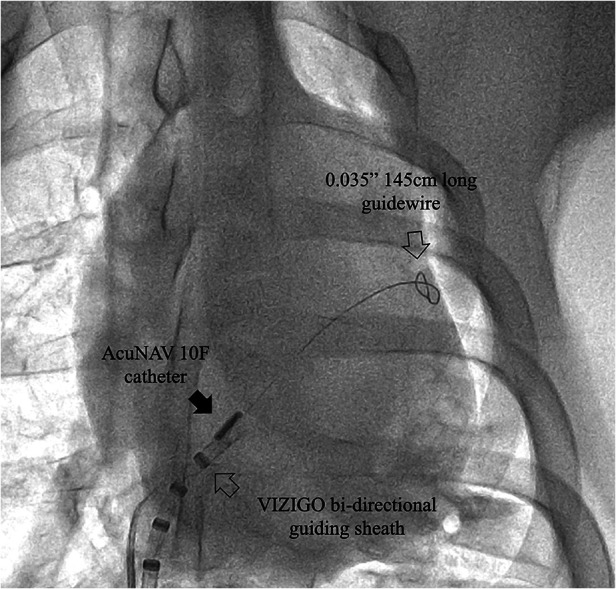
Case 0, x-ray image showing the ICE catheter positioned within the septum area. Note that the tip of the AcuNAV catheter is aligned parallel to the VIZIGO sheath and faces the site where the long guidewire passes through the atrial septum. The sheath and guidewire serve as markers in this process.

In the sheath-less trans-septal porcine model, two operators attempted to advance a commercially available ICE catheter (AcuNAV 10F) into the left atrium along a guidewire after multiple attempts. The puncture site was enlarged using an 11.5F steerable bi-directional guiding sheath (VIZIGO, Johnson and Johnson MedTech) before, and no balloon was used. It should be note that the inner diameter of the VIZIGO sheath is 8.5F and not suitable for the AcuNAV 10F catheter. For current ICE catheters, a trans-septal sheath with an inner diameter of at least 10F is required. The entire process took 33 min, including a fluoroscopy time of 18 min. Both of these durations are notably extended, considering that neither operator had prior experience with this off-label procedure (Figure 5).

**Figure 6:**
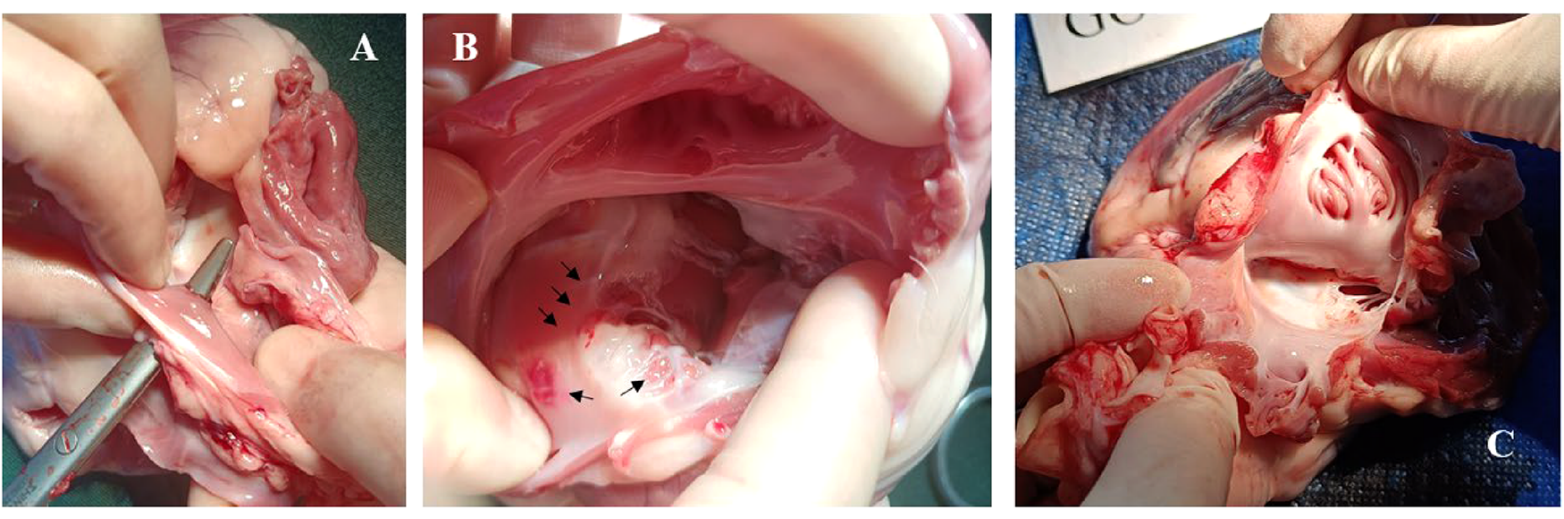
Case 0, postoperative images displayed endocardial damage resulting from an AcuNAV catheter during its passage through the septum. **(A)**, surgical forceps indicate the trans-septal puncture passway. **(B)**, multiple lesions and ecchymosis (black arrows) on the right atrium following the ICE catheter's attempt to cross the septum without a sheath. **(C)**, porcine model 4 in test group using Arch Echo™ catheter, showing normal use of an ICE catheter did not result in observable damage to the right atrium and tricuspid valve.

Postoperative cardiac anatomy examination revealed that despite the tip being softer than a typical electrophysiological catheter, the ICE catheter still caused more damage to the intima while attempting to pass through the septum (Figure 6).

### First-in-human study

On January 3, 2024, two patients were enrolled at Suzhou Kowloon Hospital. The research protocol was authorized by the ethics committee of Suzhou Kowloon Hospital and was carried out in accordance with the provisions of the Declaration of Helsinki. All patients provided written informed consent prior to participation in this study.

**Figure 7:**
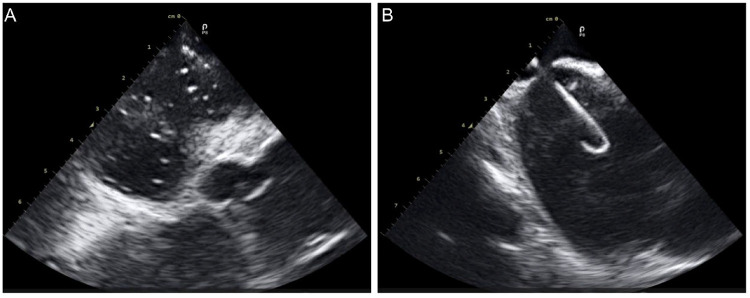
ICE images of intracardiac anatomical structures during key operational steps. **(A)**, ICE in the right atrium captured the moment of contrast agent injection; **(B)** ICE revealed the transseptal sheath successfully crossing the septum.

A 60-year-old woman with supraventricular tachycardia (SVT) and a 59-year-old woman with persistent atrial fibrillation (perAF). Both patients met the inclusion and exclusion criteria (Table 4) and had no contraindications related to ICE or radiofrequency ablation. All procedures were successfully performed using the Carto 3 system (Johnson and Johnson MedTech) in conjunction with commercially available electrophysiology catheters. The ICE catheters were utilized through Cordis 10 F short sheaths, and display ICE images of intracardiac anatomical structures during key operational steps (Figures 7A,B).

The first procedure took 220 min, while the second lasted 238 min. Fluoroscopy time was 5.2 and 12.3 min, respectively. The perAF procedure time aligned with comparable cases managed by the same operators. Beyond the initial learning curve encountered in the first case, the prolonged duration of the AV nodal reentrant tachycardia case can be attributed to two main factors: (1) This patient's left iliac artery sharply inserting into the abdominal aorta, with the angle between the aorta and the left common iliac artery being more than 90 degree; and (2) the operator's inclination towards minimizing radiation exposure.

Both patients were discharged one day after the procedure without any complications. In the post-procedure evaluation, all four operators opted for the novel ICE catheter, successfully completing the procedures with comparable imaging quality.

## Discussion

These preliminary studies indicate that a novel ICE catheter achieves a level of safety and effectiveness comparable to previously available commercial catheters. These findings highlight the potential of this novel ICE catheter to meet current clinical needs.

As mentioned earlier, there have been minimal updates to ICE catheters over the past 20 years. The primary recent catalyst for the expanded use of ICE is the rise in sophisticated anatomic interventions, including device closure, mitral annulus resizing, and electrophysiological procedures, etc (12, 13). The advantages of ICE over traditional transesophageal echocardiography makes it more convenient to use intraprocedural. Moreover, due to its angle, ICE can access more anatomically delicate areas and observe more structures, such as tricuspid valves (14, 15).

Based on the results of these FIH and *in vitro* experiments, particularly the evaluations conducted by experienced electrophysiologists and cardiac sonographers, the novel ICE catheter demonstrated its capability to successfully perform a range of cardiac interventions. The Arch Echo™ catheter has demonstrated its ability to visualize each part of different chambers. The anatomical and blood test results further indicated that, during routine operation for displaying cardiac structures, both types of ICE catheters exhibited a non-damaging impact on the internal cardiac structures. However, the thrombin time (TT) unavoidably prolonged after the administration of anticoagulant medications. The primary objective of our study was to evaluate the first use of the novel ICE catheter in human subjects, focusing on its practicality and image clarity as well as its guidance role during electrophysiology procedures. We acknowledge that, compared to existing ICE catheters, the surgical efficiency with the novel catheter may be reduced. This is expected as we are still in the initial phase of evaluating its application. As we move forward, these aspects, including operational efficiency and ICE catheter placement time, will be further explored in the upcoming registration clinical trials (Cardiac Interventional ICE Imaging Trial. NCT06344494) to provide more comprehensive data.

The scores provided by 3 experienced sonographers indicated that the performance of the two devices in the animal models was largely comparable. However, in terms of blood flow-related scores, the score of the new catheter was slightly lower, approximately by 0.1. When analyzed based on intracardiac location, there was not a significant difference observed. Considering that the operators remained consistent and the initial control group exhibited the lowest score, it suggests the presence of a learning curve during this animal testing. Notably, the test order involved the first case, followed by the last case in control group, and then the middle four test cases. Therefore, the impact of the learning curve may be most pronounced case 1. The novel Arch Echo™ catheter proven to provide patients with more options while maintaining effectiveness and safety.

Another finding of this report is that in extreme condition, the softer ICE imaging catheter can also cause damage to the endocardium. Previous studies have shown that complications from clinical cardiac electrophysiologic procedures are very low, including arterial injury (0.4%) and cardiac perforation (0.2%) (16). Recent reports on reprocessing ICE catheters have not shown increased risks (17, 18), suggesting that the complication rates may be more influenced by the catheter operating time in the cardiac chamber and off-label usage rather than reprocessing. Therefore, we recommend advancing the ICE catheter into the left atrium always within a trans-septal large inner diameter sheath (at least 10F and 81 cm usable length), although cost and availability of suitable products may be concerns at this moment. On the other hand, although accessing the left atrium provides a better view with sufficient details, especially in the left atrial appendage, a large hole in the septum can also result in additional complications and treatment. A new generation of ICE system with a wider field and larger imaging depth is expected to solve this dilemma.

The novel ICE catheter demonstrated significant maneuverability, which is crucial for performing precise cardiac interventions. Its ability to navigate through complex anatomical structures, such as the atrial septum and left atrial appendage, is a testament to its advanced design. The catheter's technical specifications, including its high spatial resolution and flexible design, allowed for detailed imaging at short distances, which is essential for accurate diagnosis and treatment planning. These findings underscore the potential of the novel ICE catheter to meet the evolving demands of modern cardiac interventions, offering a balance of maneuverability, advanced technical capabilities, and high-quality imaging.

## Conclusions

The novel ICE catheter is as safe and effective as existing products. When using the ICE catheters, operators should try to avoid off-label applications.

## Data Availability

The raw data supporting the conclusions of this article will be made available by the authors, without undue reservation.

## Supplementary material

Supplementary Video 1 Case 1, ICE in the right atrium captured the moment of contrast agent injection. *To protect the privacy of the subjects, all pertinent information is withheld.

Supplementary Video 2 Case 2, ICE revealed the transseptal sheath (SWARTZ L1, Abbott Laboratories) successfully crossing the septum. *To protect the privacy of the subjects, all pertinent information is withheld.
