# Supplementary figures and images for: First-in-human cases and preclinical experience of a novel ICE catheter
Source: Front Cardiovasc Med. 2024 Sep 10;11:1406470. doi: 10.3389/fcvm.2024.1406470 (PMC11419997; doi:10.3389/fcvm.2024.1406470)

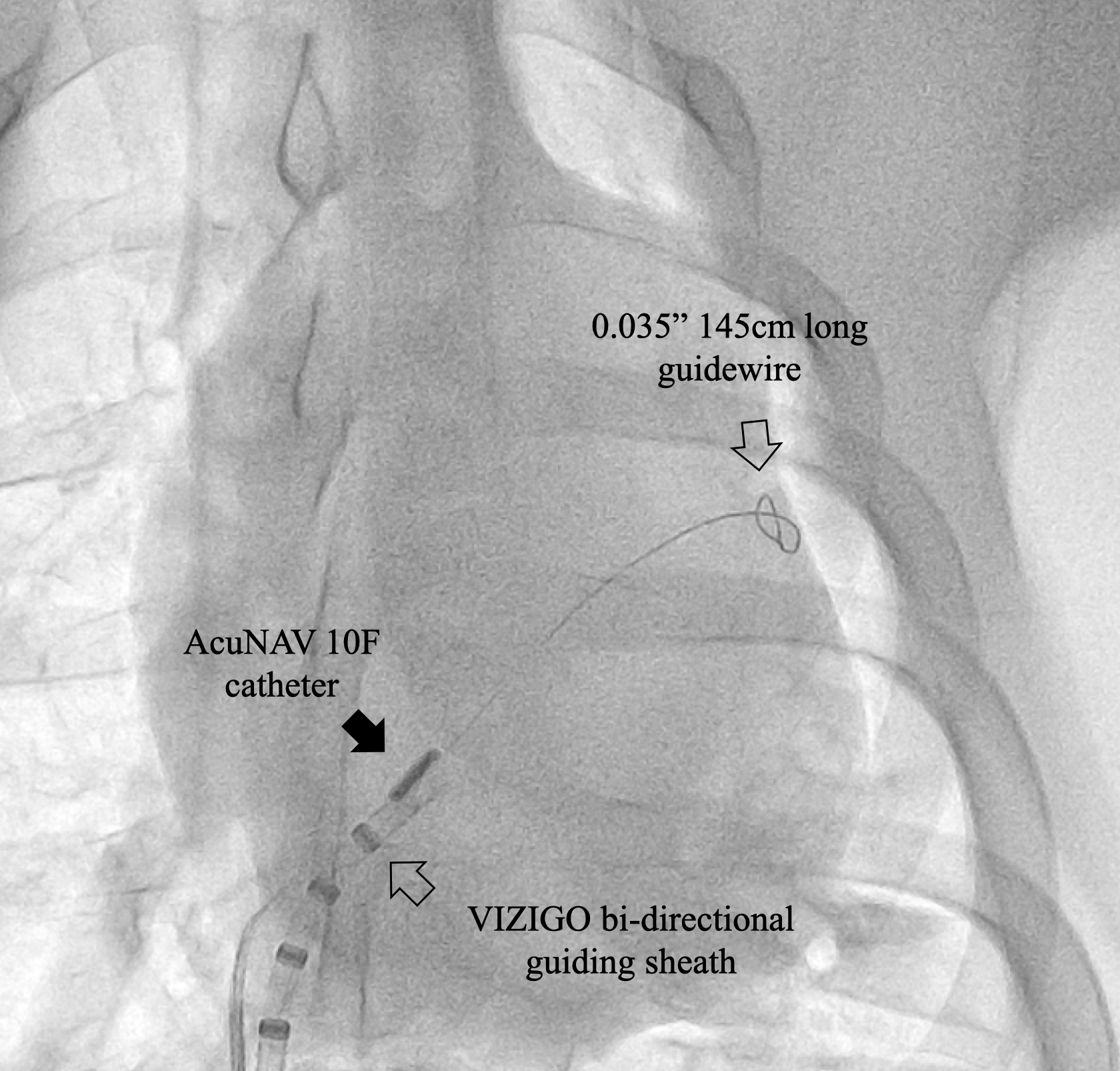

Supplement: Supplementary file 1 [file Image1.png]

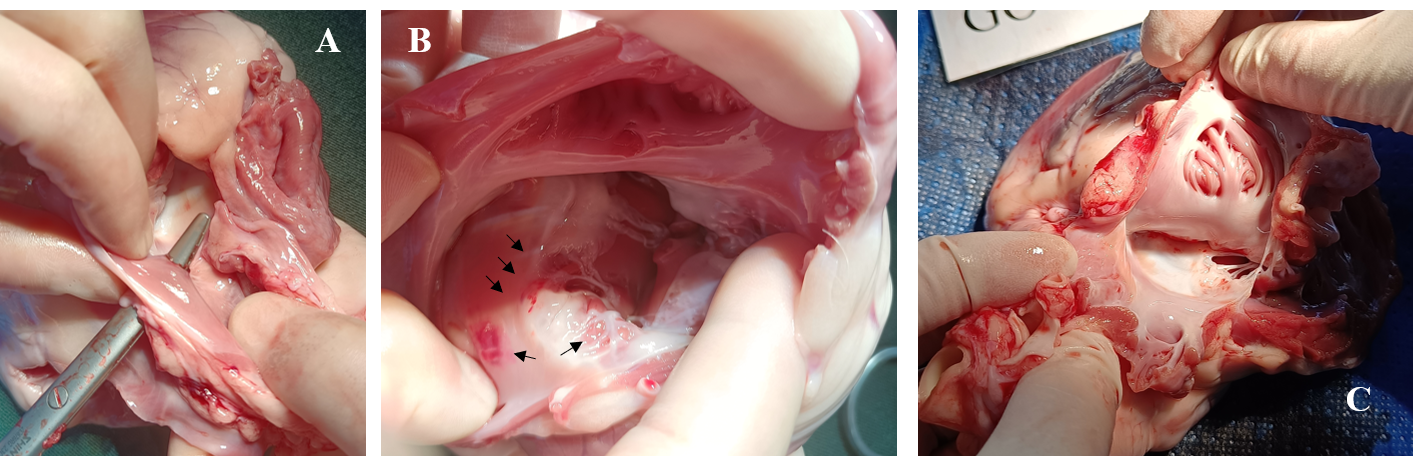

Supplement: Supplementary file 2 [file Image2.png]
